# Recipe for a Rainy Day

**Published:** 1854-12

**Authors:** 


### RECIPE FOR A RAINY DAY.

We did not intend in the conduct of our Journal to take the bread out of our own mouths by telling people how they were to get well without calling on a doctor. Still, we made no promise to that effect; we merely stated our intention, thus leaving a hole for honorable exit in case of a *change of views*, for wise men do change sometimes, fools—never. We will take care to be always consistent and always safe by being lavish in the expression of our intentions, but penuriously parsimonious in proffering promises. As a doctor, we never promised to cure certainly man, woman or child of anything, not even of the scratch of a pin, nor do we ever expect to do so silly a thing. None but the verdant or the wicked ever make promises of cure; but to the recipe "*How to lay up money for a rainy day*," as obtained from a Kennebec paper, it being kept in mind that it is healthful and agreeable to have money over and above what you owe and need.

A number of years ago, Charles and Clara S—— were married in the city of New York. Charles was wealthy and in good business—very comfortable circumstances for a young man, which tended, of course, to develope his naturally liberal disposition. Feeling thus happy and independent of the world's frowns, he proposed to his youthful bride, one day during the honey moon, to give her five thousand dollars for every "scion of his house" which should be engrafted upon the family tree —an arrangement, as may be supposed to which the lovely Clara made not the slightest objection. Time passed on,— Charles faithfully performing his agreement, and making no inquiries as to the disposition of her money by his better half, until they had been married some ten years; fortune, which had smiled with constancy, suddenly turned her back and left him apparently high and dry among the breakers of Wall street. When the crisis had arrived, he went home with a heavy heart, to announce the sad news to his wife, that he was an irretrievably ruined man—that his property had all gone to satisfy his creditors, and nothing was left. "Not exactly so bad as that, my dear," said Clara. "Wait a minute, and see what I have been doing." Thus saying, she ran up-stairs, and soon returned with a deed in her own name, for one half of an elegant block of houses in the neighborhood, worth thirty thousand dollars. "You see I have been industrious," continued she, "and have laid up something for a rainy day. If you had been as smart as your brother, we might have had the whole block, by this time!"

